# Alcohol use, cigarette smoking, vaping and number of sexual partners: A cross‐sectional study of sexually active, ethnically diverse, inner city adolescents

**DOI:** 10.1111/hex.13248

**Published:** 2021-03-28

**Authors:** Rosalie Bartholomew, Sarah Kerry‐Barnard, Nicholas Beckley‐Hoelscher, Rachel Phillips, Fiona Reid, Charlotte Fleming, Agata Lesniewska, Freya Yoward, Pippa Oakeshott

**Affiliations:** ^1^ Population Health Research Institute St George's University of London London UK; ^2^ School of Population Health and Environmental Sciences King's College London London UK; ^3^ Faculty of Medicine School of Public Health Imperial College London London UK

**Keywords:** adolescents, alcohol, cigarette smoking, risky behaviours, sexual lifestyles, vaping

## Abstract

**Context:**

There are few UK data on the prevalence and clustering of risky behaviours in ethnically diverse adolescents.

**Objectives:**

To investigate the prevalence of reported alcohol use, smoking and vaping, and explore whether these behaviours are associated with increased numbers of sexual partners.

**Design:**

Questionnaire survey of ‘Test n Treat’ chlamydia screening trial participants.

**Setting and participants:**

Sexually active students attending six London technical colleges completed confidential questionnaires and provided genitourinary samples.

**Results:**

The median age of the 509 participants was 17 years (IQR: 16‐18), 47% were male, 50% were of black ethnicity, 55% reported ≥2 sexual partners in the past year (67% of males and 45% of females) and 6.2% had chlamydia infection and 0.6% gonorrhoea. Almost half (48%) reported getting drunk in the past month, 33% smoked cigarettes and 7% had ever vaped.

A larger percentage of students with ≥2 sexual partners than 0‐1 partners reported getting drunk in the past month (53.7%, 144/268% versus 42.2% 94/223, adjusted prevalence ratio: 1.33, 95% confidence interval: 1.11‐1.61) and smoking cigarettes (36.6%, 100/273% versus 30.2%, 67/222, 1.34 (1.05‐1.70)). By contrast, multiple sexual partners were not associated with vaping or chlamydia infection, but numbers were small.

**Conclusions:**

We found high prevalences of risky behaviour and an association between multiple sexual partners and smoking and/or getting drunk. Findings support the introduction of compulsory sex and relationship education in UK secondary schools, including information about the adverse effects of alcohol and smoking.

**Public contribution:**

Participants helped with study design, conduct and interpretation.

**Abbreviations**
CI: confidence interval
PR: prevalence ratio
STI: sexually transmitted infection

## 1 BACKGROUND

There is on‐going concern in many countries that increasing numbers of young adults undertake risky behaviours including getting drunk, cigarette smoking, vaping (using electronic cigarettes) and having multiple sexual partners.^1^, ^2^, ^3^ In addition, many reports show that risky behaviours tend to cluster together.^1^, ^2^, ^4^ A population‐based survey of British young people aged 16‐24 found participants reporting weekly or more frequent binge drinking (≥ weekly) or recent drug use (within the past 4 weeks) were more likely to report one or more new sexual partners in the past year.^5^ Similarly in Italian adolescents, lifetime cannabis use, high alcohol intake and early sexual intercourse were more frequent among heavy smokers.^4^ However, there are few UK data on the extent of clustering of risky behaviours, specifically in inner‐city, ethnically diverse adolescents^6^, ^7^; there have been calls for more studies of substance use and sexual behaviour in teenagers.^2^, ^5^, ^8^ Adolescence is a key stage in life when unhealthy habits are most often established,^9^ and these can lead to health inequalities later in life.^1^, ^10^

Using cross‐sectional, baseline data from the ‘Test and treat’ (TnT) feasibility trial (which explored the uptake of rapid chlamydia tests and same‐day on‐site treatment^11^), we examined the prevalence of reported alcohol use, smoking (including heavy smoking defined as ≥10 cigarettes daily) and vaping, and explored possible associations with numbers of sexual partners (≥2 versus 0‐1) within the past year.

## 2 METHODS

### 2.1 Participants

In September‐October 2016, we recruited 509 sexually experienced male and female students aged 16‐24 years. We approached students in public areas at six inner London further education/technical colleges^11^ and asked whether they would consider helping us in a study on sexual health. Students who had never had sexual intercourse were ineligible. Following informed consent, students completed a confidential baseline questionnaire and supplied a self‐taken genitourinary sample for later chlamydia/gonorrhoea testing using the Cobas 4800 CT/NG system (Roche Diagnostics), with positives confirmed using the Cepheid CT/NG GeneXpert system. Participants were given £5.

The questionnaire asked about date of birth, gender and ethnic origin. It also asked the following:

Altogether in the last year, how many people have you had sexual intercourse with? One, Two, Three or more, None.

In the past month, how many times have you got drunk due to alcohol? 1‐4, 5 or more, None.

How many cigarettes do you smoke each day? More than 10, 1‐10, None.

Do you vape (use electronic cigarettes)? Yes, No.^5^, ^11^

### 2.2 Participant involvement

Four students on the trial steering committee gave advice throughout the study, 26 took part in qualitative interviews,^12^ and findings were fed back to participants for their comments during an end of the study workshop.

### 2.3 Statistical analysis

The sample size was determined to give adequate precision in the feasibility outcomes of the TnT study.^11^ Statistical analyses were performed with STATA 10. The prevalence of getting drunk, smoking, vaping and multiple partners was presented as percentages with 95% confidence intervals (CIs). We used prevalence ratios (PRs) to explore possible associations between number of sexual partners (≥2 versus 0‐1) and getting drunk, smoking or vaping at baseline. Adjusted analyses were performed using multivariable Poisson models with a robust variance estimator, in order to model the prevalence ratio explicitly whilst stabilizing the variance.^13^ Sensitivity analyses using log‐binomial regression models were also undertaken and gave similar results.

Earlier studies suggested that the prevalence of risky behaviours may vary by ethnic group,^5^, ^14^ and higher rates of risky sexual behaviour in males than females.^5^, ^7^, ^15^ Therefore, we adjusted multivariable analyses for two binary covariates: gender (male/female) and white ethnicity (yes/no).

## 3 RESULTS

### 3.1 Characteristics of participants

The median age of the 509 eligible participants was 17 years (IQR: 16‐18), and 47% were male (Table 1). Half of the participants (50%, n = 252) described their ethnic origin as black (black African/black Caribbean/black other), 26% (n = 133) white, 14% (n = 69) Mixed/multiple ethnic groups, 6% (n = 28) Asian/Asian British and 4% (n = 22) other. The prevalence of *Chlamydia trachomatis* infection was 6.2% (31/503) and *Neisseria gonorrhoeae* 0.6% (3/503). Of 503 responders, over half said they always (36%) or usually (19%) used condoms. Around a third (36%) said they had attended a health‐care facility for sexual health reasons in the past 6 months.

**TABLE 1:** Characteristics of 240 male and 269 female participants

	Male (n = 240)	Female (n = 269)
Total	47% (240/509)	53% (269/509)
White ethnicity	24% (58/239)	28% (75/265)
Mixed/multiple ethnic groups	15% (35/239)	13% (34/265)
Asian	8% (20/239)	3% (8/265)
Black/African/Caribbean	49% (118/239)	51% (134/265)
Any other ethnic group	3% (8/239)	5% (14/265)
Median age in years	18.1	17.8
How many times got drunk in the past month		
None	52% (123/235)	51% (135/265)
1‐4	35% (82/235)	34% (89/265)
≥5	13% (30/235)	16% (41/265)
How many cigarettes smoked a day		
None	71% (168/238)	64% (337/266)
1‐10	26% (62/238)	28% (74/266)
>10	3% (8/238)	9% (23/266)
Ever use of vaping (electronic cigarettes)		
Yes	9% (20/236)	7% (17/261)
No	92% (216/236)	94% (244/261)
Number of sexual partners in past year		
None	4% (9/236)	5% (13/263)
One	30% (70/236)	50% (132/263)
Two	22% (51/236)	28% (73/263)
Three or more	45% (106/236)	17% (45/263)
Chlamydia at baseline	7% (16/236)	6% (15/267)
Gonorrhoea at baseline	1% (3/236)	0% (0/267)

### 3.2 Alcohol use

Overall 48.4% (242/500, 95% CI: 44.0%‐52.8%) of participants reported getting drunk on one or more occasions in the past month including 14% (71) reporting more than five occasions.

### 3.3 Cigarette smoking

Of 504 responders, 33.1% (167, 95% CI: 29.0%‐37.2%) said they smoked cigarettes with 27.0% (136) smoking 1‐9 cigarettes a day and 6.2% (31) 10 or more a day.

### 3.4 Vaping

Ever use of e‐cigarettes was reported by 7.4% (37/497, 95% CI: 5.1%‐9.8%) of participants.

### 3.5 Sexual partners and risk behaviours

Of 499 responders, 55.1% (275, 95% CI: 50.8%‐59.5%) reported that they had two or more sexual partners in the past year. Reporting multiple sexual partners was more common in males than females (66.5% vs 44.9%, prevalence ratio: 1.48 [95% CI: 1.26‐1.74]) and in those of non‐white rather than white ethnicity (58.0% vs 48.1%, 1.21 (0.99‐1.47)). After adjusting for gender and ethnicity, reporting at least one episode of being drunk in the past month or being a regular cigarette smoker was more common in those reporting ≥2 sexual partners in the past year than those with 0‐1 partners (Table 2). This did not apply to vaping or heavy smoking, but numbers were small.

**TABLE 2:** Comparison of the prevalence of various lifestyle factors in young people with ≥2 sexual partners versus 0‐1 sexual partners in the past year

Lifestyle factor	≥2 sexual partners in the past year	0‐1 sexual partners in the past year	Unadjusted prevalence ratio (95% confidence interval)	Adjusted prevalence ratio^a^ (95% confidence interval)
At least one episode of being drunk in the past month	53.7% (144/268)	42.2% (94/223)	1.27 (1.05‐1.54)	1.33 (1.11‐1.61)
Regular cigarette smoker	36.6% (100/273)	30.2% (67/222)	1.21 (0.94‐1.56)	1.34 (1.05‐1.70)
Ever vaped‐smoked electronic cigarettes	7.1% (19/268)	8.1% (18/221)	0.87 (0.47‐1.62)	0.89 (0.45‐1.76)
Heavy smoker (10+ per day)	5.5% (15/273)	7.2% (16/222)	0.76 (0.39‐1.51)	0.96 (0.49‐1.87)

^a^ Adjusted for gender (male/female) and ethnicity (white/other ethnic group).

### 3.6 Chlamydia infection and risky behaviours

Chlamydia was not significantly associated with any risky behaviour, but numbers of infections were relatively small. The prevalence of chlamydia in those reporting getting drunk was 4.6% (11/240) versus 8% (20/254) in the remainder. Chlamydia prevalence was 7% (12/167) in smokers versus 6% (19/332) in non‐smokers; and 0% (0/36) in those who vaped versus 7% (31/455) in those who had never vaped. In those reporting ≥2 partners in the past year, chlamydia prevalence was 7% (20/273) versus 5% (11/220) in those with 0‐1 partner.

### 3.7 Participant feedback

Student attenders at the end of the study workshop confirmed that most had received little education about risky sexual behaviours. Sex and relationship education was ‘mainly about putting on condoms…….not to get pregnant’.

## 4 DISCUSSION

### 4.1 Principal findings

Almost half of these young, multi‐ethnic students reported getting drunk in the past month and a third were smokers, with both behaviours more common in those reporting multiple sexual partners. Only 7% reported ever using e‐cigarettes. There were no clear associations between risky behaviours and chlamydia infection, but numbers were small.

### 4.2 Strengths and limitations

This is the largest UK community–based study of alcohol, smoking and sexual lifestyles in high‐risk, inner‐city adolescents. It included a high number of adolescents from ethnic minority groups, notably 118 young black males who are an under‐researched, hard‐to‐reach population who may not often attend primary health‐care services. In addition, our study provides novel information on risky behaviours in urban teenagers, including ever use of vaping.

There are several limitations. Data were cross‐sectional so we cannot say anything about causality.^5^ There may be information/recall and/or social desirability bias as we used self‐reported behaviours. Adolescents may not be reliable historians. (This may partly explain the differences between the genders in reported numbers of sexual partners.^5^, ^7^) There may be selection bias as we only recruited from six colleges, and we do not know how representative these adolescents are compared with other sexually active young people from ethnically diverse urban areas. For example, half of the participants described themselves as being of black ethnicity. Data from the Office for National Statistics show that in 2018 21% of students at English further education/technical colleges were from black and ethnic minority groups, but this proportion is higher in inner London.

We only recruited students who said they were sexually active, and this may vary by culture. However, the association between male gender and risky sexual behaviour (such as having more sexual partners) is in line with other studies.^5^, ^15^ Other weaknesses are that we did not define what we meant by ‘getting drunk’ or ‘sexual intercourse’, nor did we ask about socio‐economic status which may be associated with risky behaviour.^1^ In addition, we could not look at students who had dropped out of classes at the colleges, or adolescents classified as ‘NEETs’ (Not in Education, Employment or Training). These could be young people with more risky behaviours. Although we did not include those with special educational needs, we did include some participants with lower literacy levels who asked for help in completing the questionnaires. As vaping is a relatively new risk behaviour, we asked about ever vaping but used different timing for alcohol (drunk in past month) and smoking (cigarettes per day). It is likely that the rate of ever vaping in adolescents will increase over time.^2^, ^16^

### 4.3 Comparison with other studies

Our data are in line with other studies from the United States, Canada and the UK showing high rates of alcohol misuse in adolescents, and a relationship between this and an increased number of sexual partners [1, 2, 8].^3^ However, none of these studies included such a high proportion of adolescents from black and minority ethnic groups who may be less likely than older white individuals to be surveyed. Rates of smoking were similar to other studies in comparative age groups.^2^ However, a large 2010‐12 study of British young people aged 18‐24 found a slightly higher reported ever use of vaping: 12% versus 7% in our study.^16^ Our participants were younger and more ethnically diverse, and 90% were adolescents. A Swedish study of 2185 teenagers found reported e‐cigarette use was associated with personal and parental tobacco use, physical inactivity and an unhealthy diet.^8^ We did not collect data on all these variables. Most recently, the rise in adolescent vaping in the United States and Canada shown in cross‐national surveys has led to the consideration of measures to protect young people. These include greater restrictions on advertising, flavours and retail access to e‐cigarettes.^2^ Finally, the prevalence of chlamydia in our participants was almost double the national average in 16‐ to 24‐year‐olds (367.5/100 000) reported by Public Health England in 2017. This supports our current findings on lifestyles, which also suggest this group may have high rates of risky behaviours.^17^

### 4.4 Implications for policy and practice

Findings could influence clinical practice and public health policy. We found high prevalences of getting drunk, smoking and multiple sexual partners in sexually experienced, teenage further education college students in London with possible associations between getting drunk, cigarette smoking and increased number of sexual partners. These findings support calls for more education and health promotion for similar hard‐to‐reach, young or multi‐ethnic populations.^5^ However, there may be limited visible intervention at the college level that targets such behaviours with tutors reporting that sex education on‐site was very much ‘hit and miss’. Interviews with 26 student participants^12^ showed very low levels of knowledge about sexually transmitted infections with only 15% aware that chlamydia can cause infertility. In addition, there were suggestions that some teenagers considered themselves invulnerable to sexually transmitted infections despite engaging in risky behaviours.^12^ Young people's lack of knowledge about risky behaviours and sexually transmitted infections is also a problem across Europe, the United States and Australia.^11^, ^17^, ^18^

Engagement in multiple risk behaviours in adolescence is an important driver of health inequalities in later life.^1^ It has been suggested that early life interventions based in schools could help prevent such outcomes.^1^ In line with recommendations from Italy^4^ and elsewhere,^5^ the UK Department of Health has highlighted the potential benefits of adopting an integrated approach to health improvement by addressing behaviours such as substance use together with sexual behaviours. This could be provided by specialist teachers, school nurses or general practitioners backed by online publicity, information packs and improved personal, social and health education lessons. Although we provided online and paper educational fliers and posters, future studies might explore the impact of more interactive education at the time of testing on future behaviour. In addition, small financial incentives for testing might encourage uptake and reduce stigma as people could say they were just getting tested for the money.^19^ In 2020, the UK government made sex and relationship education compulsory in state secondary schools.

## 5 CONCLUSIONS

In the first UK community–based study of risky behaviours in sexually active, inner‐city adolescents, we found high rates of reporting multiple partners (55%), getting drunk in the past month (48%) and cigarette smoking (33%). Participants with ≥2 partners in the past year were more likely than those with 0‐1 partners to have got drunk in the past month and to smoke cigarettes. General practitioners doing health promotion in potentially vulnerable teenagers could consider asking about more than one risky behaviour. In addition, sex education for adolescents should be compulsory and address both sexual behaviours and substance use.^1^, ^5^, ^10^ Policymakers should be aware of the urgent need for better health education for inner‐city teenagers.

## CONFLICTS OF INTEREST

Pippa Oakeshott is a member of the NIHR South London Collaboration for Leadership in Applied Health Research and Care, and of the *e*STI^2^ consortium funded under the UKCRC Translational Infection Research Initiative. Fiona Reid is supported by the National Institute for Health Research (NIHR) Biomedical Research Centre based at Guy's and St Thomas' NHS Foundation Trust and King's College London. The other authors declare that they have no competing interests.

## AUTHORS' CONTRIBUTIONS

RB, PO, FR and SKB designed the study. AL did the chlamydia tests, and RB, SKB, N B‐H, RP and FR analysed the data. RB wrote the first draft of the paper to which all authors including CF and FY then contributed. All authors read and approved the final manuscript.

## ETHICS APPROVAL AND CONSENT TO PARTICIPATE

Bromley Research Ethics Committee reviewed the study (Reference Number 15/LO/1929). Participants gave written informed consent.

## Data Availability

Data are available from NBH subject to appropriate approvals.
